# Comparative transcriptome analysis of the antenna and proboscis reveals feeding state-dependent chemosensory genes in *Eupeodes corollae*

**DOI:** 10.1098/rsob.230208

**Published:** 2024-01-10

**Authors:** Ruipeng Chen, Dong Ai, Guirong Wang, Bing Wang

**Affiliations:** ^1^ State Key Laboratory for Biology of Plant Diseases and Insect Pests, Institute of Plant Protection, Chinese Academy of Agricultural Sciences, Beijing 100193, People's Republic of China; ^2^ Shenzhen Branch, Guangdong Laboratory for Lingnan Modern Agriculture, Genome Analysis Laboratory of the Ministry of Agriculture, Agricultural Genomics Institute at Shenzhen, Chinese Academy of Agricultural Sciences, Shenzhen 518120, People’s Republic of China

**Keywords:** *Eupeodes corollae*, olfactory plasticity, mRNA sequencing, chemosensory-related genes, feeding state

## Abstract

The physiological state of an insect can affect its olfactory system. However, the molecular mechanism underlying the effect of nutrition-dependent states on odour-guided behaviours in hoverflies remains unclear. In this study, comparative transcriptome analysis of the antenna and proboscis from *Eupeodes corollae* under different feeding states was conducted. Compared with the previously published antennal transcriptome, a total of 32 novel chemosensory genes were identified, including 4 ionotropic receptors, 17 gustatory receptors, 9 odorant binding proteins and 2 chemosensory proteins. Analysis of differences in gene expression between different feeding states in male and female antennae and proboscises revealed that the expression levels of chemosensory genes were impacted by feeding state. For instance, the expression levels of *EcorOBP19* in female antennae, *EcorOBP6* in female proboscis, and *EcorOR6*, *EcorOR14*, *EcorIR5* and *EcorIR84a* in male antennae were significantly upregulated after feeding. On the other hand, the expression levels of *EcorCSP7* in male proboscis and *EcorOR40* in male antennae were significantly downregulated. These findings suggest that nutritional state plays a role in the adaptation of hoverflies' olfactory system to food availability. Overall, our study provides important insights into the plasticity and adaptation of chemosensory systems in hoverflies.

## Graphical abstract

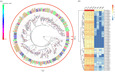

## 1. Introduction

The chemosensory system is essential for insects to identify chemical signals during feeding, mating and oviposition [1,2], and antennae and proboscises are vital organs in olfactory and gustatory perception [3–8]. Various sensilla are distributed on these chemosensory organs, and they house the dendrites of olfactory sensory neurons. At the molecular level, several chemosensory-related proteins play a crucial role in insect olfactory detection, including odorant receptors (ORs), gustatory receptors (GRs), ionotropic receptors (IRs), odorant binding proteins (OBPs), chemosensory proteins (CSPs) and sensory neuron membrane proteins (SNMPs) [9–12]. The proposed molecular mechanism for insect olfactory detection involves binding of OBPs to chemical signals that then travel to ORs expressed on the dendritic membrane of the olfactory sensory neurons. When ORs are activated, chemical signals are converted into electrical signals that travel to the central nervous system [13].

Olfactory plasticity is a change in olfactory-guided behaviour in response to the same stimulus due to modulation within the sensory system [14]. Plasticity is a crucial factor in the survival of insects, and it can be influenced by multiple factors, which cause changes in plasticity by inducing changes in both the peripheral and central nervous systems [14,15]. Physiological state, biotic and abiotic factors and experience-induced alterations can all affect olfactory and gustatory plasticity [16]. For example, nutrition-dependent state can affect odour-guided behaviours in insects [14]. In *Drosophila melanogaster*, food odours trigger the host-seeking behaviour of starved individuals, which involves changes in both peripheral neuron responses and brain neuron integration [16,17]. Mosquitoes, on the other hand, suppress host-seeking behaviours after a blood meal and become more sensitive to odours related to oviposition sites [18,19]. The expression levels of specific chemosensory-related genes in the peripheral chemosensory system can influence olfactory and gustatory plasticity, affecting odorant detection [14,16]. In *D. melanogaster*, for instance, the expression level of *DmelOBP99* sharply increases after 24 h of starvation, influencing ingestive behaviours [20,21]. Furthermore, in *Culex quinquefasciatus* there are differences in the expression of olfactory genes in antennae following a blood meal [22,23]. This study indicates that the peripheral nervous system does indeed change with variations in the nutritional status of insects.

Hoverflies (Diptera: Syrphidae) are beneficial insects in agroecosystems, serving as natural enemies in their larval stage and as pollinators in the adult stage [24,25]. Hoverfly larvae consume vast numbers of aphids, while the adults typically feed on nectar, which serves as a nutrient source, and pollen, which contributes to sexual maturity, from a variety of plants [26]. Adult hoverflies rely on a sophisticated chemosensory system to detect various chemical cues and locate potential food sources [27–30]. The sexual maturation of adults following feeding may alter the expression levels of chemosensory-related genes and neuromodulators, thereby impacting mating behaviour. However, the molecular mechanism underlying the effect of nutrition-dependent state on odour-guided behaviours in hoverflies remains unclear.

In previous work, we identified a large number of chemosensory genes in the antennal transcriptomes of *Episyrphus balteatus* and *Eupeodes corollae* (Diptera: Syrphidae) [6]. In this study, we aimed to investigate the impact of changes in the physiological state of *E. corollae* on chemosensory plasticity in the peripheral nervous system. We identified chemosensory-related genes from the transcriptomes of the antennae and proboscises of *E. corollae* under different nutritional states and analysed the genes that were differentially expressed depending on feeding state. To test whether feeding state can alter the expression levels of these genes, we performed real-time quantitative PCR (qPCR) to examine selected differentially expressed genes (DEGs). Our study sheds light on the potential roles of chemosensory-related genes in the olfactory plasticity of *E. corollae*.

## 2. Material and methods

### 2.1. Insect rearing and tissue collection

*Eupeodes corollae* adults were maintained at the Institute of Plant Protection, Chinese Academy of Agricultural Sciences (Beijing, China), under the following conditions: 26 ± 1°C, 60% relative humidity, and a photoperiod of 14 h light and 10 h darkness. Larvae were reared with the aphid *Acyrthosiphon pisum* on broad bean plants (*Vicia faba* L.). Newly eclosed male and female adults were kept separately in isolated cages to prevent mating. Individuals of each sex were randomly divided into feeding and starvation groups. The feeding group (treatment group) was provided with a 10% honey solution and pollen, while the starvation group (control group) was only given distilled water. Antennae and proboscises of 3- to 4-day-old adults of both sexes were collected and stored at −80°C. The eight groups were as follows: female antennae from control (FAC), female antennae from treatment (FAT), female proboscises from control (FPC), female proboscises from treatment (FPT), male antennae from control (MAC), male antennae from treatment (MAT), male proboscises from control (MPC) and male proboscises from treatment (MPT). There were three biological replicates for each group.

### 2.2. cDNA library construction and sequencing

Total RNA was isolated from antennae and proboscises using TRizol Reagent (Invitrogen, Carlsbad, CA, USA) following the manufacturer's instructions [6]. The quality of total RNA was verified using an Agilent 2100 Bioanalyzer (Agilent RNA 6000 Nano Kit) and a NanoDrop ND-2000 spectrophotometer (NanoDrop Products, Wilmington, DE, USA). Ten micrograms of total RNA from each sample were used to construct cDNA libraries for subsequent sequencing. RNA sequencing (RNA-seq) libraries were constructed and sequenced on the Illumina HiSeq X Ten platform (San Diego, CA, USA) with a paired-end (PE150 bp) strategy at Beijing Genomics Institute (BGI, Shenzhen, China). The raw sequence data (accession number: PRJNA791698) were uploaded to the Short Read Archive (SRA) database of the National Center for Biotechnology Information (NCBI; https://www.ncbi.nlm.nih.gov/sra).

### 2.3. Assembly and functional annotation

Low quality reads and adaptor sequences were removed from raw reads using SOAPnuke software (v.1.4.0; https://github.com/BGI-flexlab/SOAPnuke) [31]. Clean reads in FASTQ format were de novo assembled using Trinity (v.2.5.1) with default parameters [32]. Then, TGIR Gene Indices clustering tools (TGICL) was used to cluster and delete redundant transcripts [33]. For several samples, the unigenes of each library were submitted to the TGICL program again to create final unigenes for functional and expression analysis. For functional annotation of unigenes, unigene sequences were used as queries in BLAST (v.2.2.23) searches against the Nt, Nr, Pfam, KOG, Swissprot and KEGG databases using an *E*-value ≤ 1 × 10^−5^ as the threshold [34]. The BLAST results were then imported into the Blast2GO (v.2.5.0) software for gene ontology (GO) annotation [35].

### 2.4. Identification of candidate chemosensory genes

Candidate unigenes encoding putative chemosensory genes (*ORs*, *IRs*, *GRs*, *OBPs*, *CSPs* and *SNMPs*) were identified from the transcriptome assembly and GO annotations [6]. Chemosensory-related keywords were used to extract candidate genes from the annotation. To identify candidate unigenes, tBLASTn searches were also performed with an *E*-value ≤ 1 × 10^−5^ as a threshold using available olfactory proteins from Diptera species as queries. Next, the redundant sequences were removed using the ContigExpress module of Vector NTI 11.0 (Invitrogen InforMax, Inc., USA).

### 2.5. Sequence alignment and phylogenetic analysis

The open reading frames (ORFs) of candidate chemosensory genes were manually verified using ORF Finder (https://www.ncbi.nlm.nih.gov/orffinder). The N-terminal signal peptides of putative OBPs and CSPs were predicted using the default parameters of the SignalP 4.1 server (https://services.healthtech.dtu.dk/service.php?SignalP-4.1) [36,37]. Transmembrane domains of candidate GRs and IRs were predicted using TMHMM 2.0 [38] (https://services.healthtech.dtu.dk/service.php?TMHMM-2.0). Nucleotide sequences of candidate chemosensory genes were translated into amino acid sequences with Expasy (https://web.expasy.org/translate/) and aligned with Muscle (v.3.8.31) [39].

The alignments of all amino acid sequences were performed using MAFFT (v.7.164b) [40]. The phylogenetic trees of CSPs were constructed using RaxML v.8 with the Jones–Taylor–Thornton (JTT) model based on 1000 bootstrap replications [41]. The OR dataset contained sequences from *E. balteatus* [6], *D. melanogaster* [42,43], *Musca domestica* [44] and *Bactrocera dorsalis* and *Calliphora stygia* [45]. The GR dataset contained sequences from *E. balteatus* [6], *D. melanogaster* [46], *B. dorsalis* and *C. stygia* [45] and *M. domestica* [44]*.* The IR dataset contained sequences from *E. balteatus* [6], *D. melanogaster* [47,48], *C. stygia* [45] and *Anopheles gambiae* [48]*. Eupeodes corollae* OBPs were compared to sequences from *E. balteatus* [6], *D. melanogaster* [49], *B. dorsalis* and *C. stygia* [45] and *M. domestica* [44]. *Eupeodes corollae* CSPs were compared to sequences from *E. balteatus* [6], *D. melanogaster* [49], *C. stygia* [45] and *A. gambiae* [50]*. Eupeodes corollae* SNMPs were compared to sequences from *E. balteatus* [6], *D. melanogaster* [51,52] and *A. gambiae* [52]. The sequences of *C. stygia* and *E. balteatus* were obtained from transcriptome data and other sequences were obtained from genomic data (electronic supplementary material, table S1). The phylogenetic trees were viewed and edited using FigTree v.1.4.4 (http://tree.bio.ed.ac.uk/software/figtree).

### 2.6. Differentially expressed gene analysis

The Bowtie2 (v.2.2.5) software was used to map clean reads to the unigenes, and RSEM (v.1.2.12) was used to calculate expression levels of unigenes in each sample [53,54]. The heatmap plots of gene expression levels were generated by the R package pheatmap (v.1.0.12). The package DESeq2 was used for DEG analysis based on the negative binomial distribution [55]. The volcano map of differential gene expression was constructed using the R package ggplot2 (v.3.3.5) with the criteria for differential expression set at |log(fold change)| ≥1 and corrected FDR ≤ 0.05.

### 2.7. Expression level analysis using real-time qPCR

Real-time qPCR analysis was performed to verify the expression of candidate chemosensory genes. The primers used in real-time qPCR were designed using Beacon Designer 8.14 (PREMIER Biosoft International, USA) software and are listed in electronic supplementary material, table S2. cDNA was synthesized using the EasyScript All-in-One First-Strand cDNA Synthesis SuperMix for qPCR (TransGen Biotech Co. Ltd, Beijing, China) according to the manufacturer's protocol. The real-time qPCR analysis was performed using PerfectStart Green qPCR SuperMix (TransGen Biotech Co. Ltd) on the QuantStudio 6 flex system (Thermo Fisher Scientific, Applied Biosystems, Waltham, MA, USA) with the following programme: initial denaturation at 94°C for 2 min, followed by 40 cycles of 94°C for 5 s, 60°C for 34 s and 72°C for 10 s. Melting curve analysis was performed after the amplification programme to verify the specificity of each primer pair. Each reaction was performed in three biological replicates with negative controls. To obtain more accurate data, the elongation factor (*EF*) gene and ribosomal protein S3 (*RPS3*) gene were selected as the reference genes for normalizing the expression results. The stable expression of *EF* and *RPS3* across different tissues and treatments was evaluated by Normfinder software [56] (electronic supplementary material, table S3).

The cDNA was serially diluted by a factor of 2, and the standard curve for each gene was generated using at least five concentrations to ensure that the amplification efficiency of the individual genes ranged from 90% to 110%. The 2^−ΔΔCt^ method was used to calculate the relative expression levels [57]. Each reaction was performed in three biological replicates. The differences in gene expression between the treatment and control were analysed with Student's *t*-test (*α* *=* 0.05) using SPSS 25.0 software (SPSS Inc., Chicago, IL, USA), and data were plotted using GraphPad Prism 8.0.1 software (GraphPad Software Inc., San Diego, CA, USA; www.graphpad.com).

## 3. Results

### 3.1. Transcriptome assembly and functional annotation

Sequencing of the antennal and proboscis transcriptomes of male and female hoverflies fed honey and pollen (feeding group) or water (starvation group) (three biological replicates each) yielded a total of 1553.1 million raw reads*.* After removing the low-quality reads, approximately 1543.44 million clean reads from all 24 tissue samples were obtained, and there were as many as 64.31 million clean reads per tissue with a Q30 percentage greater than 89% (electronic supplementary material, table S4). These clean reads were assembled into 74 715 non-redundant unigenes with a total length of 116 131 249 bp. The average unigene length was 1554 bp and the N50 was 3126 bp (electronic supplementary material, table S5).

The unigenes were then annotated by performing searches against seven major functional databases. The numbers of genes annotated using each database were 42 474 (Nr: 56.85%), 17 162 (Nt: 22.97%), 31 528 (Swiss-Port: 42.20%), 31 263 (KOG: 41.84%), 31 466 (KEGG: 42.11%), 20 876 (GO: 27.94%) and 30 670 (PFAM: 41.05%) (electronic supplementary material, table S6). The distribution of GO terms was similar between samples. The most abundant sub-categories were ‘cellular process’, ‘metabolic process’ and ‘regulation of the biological process' for the biological process category, ‘cell’, ‘cell part’ and ‘membrane’ for the cellular component category and ‘binding’, ‘transporter activity’ and ‘catalytic activity’ for the molecular function category (electronic supplementary material, figure S1*a*). Analysis of the species distributions of unigenes showed that 12.47% of unigenes had matches to *Galendromus occidentalis*, 8.40% to *Rhagoletis zephyrs*, 8.2% to *Lucilia cuprina* and 6.03% to *M. domestica* (electronic supplementary material, figure S1*b*).

### 3.2. Identification of chemosensory-related genes differentially expressed under different feeding states

#### 3.2.1. Odorant receptors

**Figure 1.**
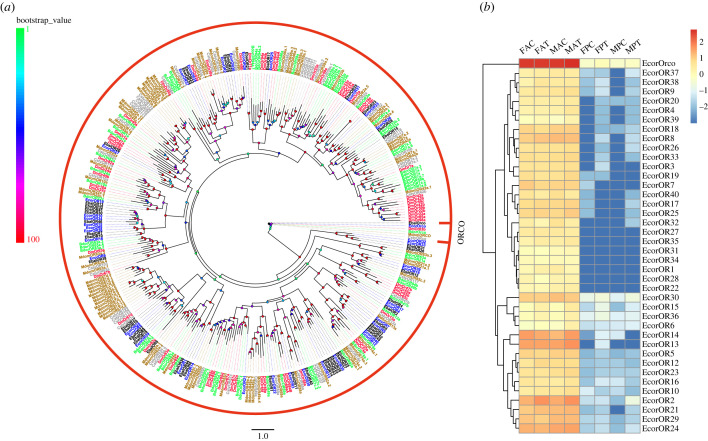
Phylogenetic tree and heatmap of ORs. (*a*) Phylogenetic tree constructed using OR protein sequences from *E. corollae* (blue), *E. balteatus* (black), *D. melanogaster* (red), *B. dorsalis* (green), *C. stygia* (grey) and *M. domestica* (brown). The phylogenetic tree was rooted using Orco orthologues, and bootstrap values are shown. (*b*) Heatmap of *OR* gene expression in the antenna and proboscis. The expression values are represented as mean values, and the data were normalized as follows: log_10_(FPKM + 0.001). FAC, female antennae from control; FAT, female antennae from treatment; FPC, female proboscis from control; FPT, female proboscis from treatment; MAC, male antennae from control; MAT, male antennae from treatment; MPC, male proboscis from control; MPT, male proboscis from treatment. Control is *E. corollae* fed with ddH_2_O, and treatment is *E. corollae* fed with 10% honey and pollen.

**Figure 2.**
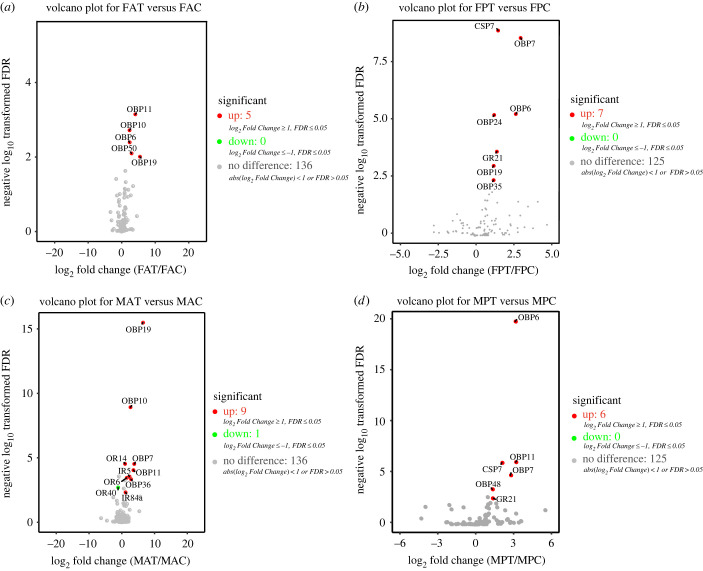
Volcano plots of the chemosensory DEGs in the antenna and proboscis under different feeding states. (*a–d*) DEGs between feeding and starvation treatments in female antennae (*a*), female proboscises (*b*), male antennae (*c*) and male proboscises (*d*). Grey dots represent genes that did not meet the FDR threshold of 0.05, and red and green dots represent genes that were significantly up- and downregulated, respectively, in the different treatment groups.

We identified 40 *ORs* in the antenna and proboscis transcriptomes of *E. corollae*, 23 of which had full-length ORFs. No additional ORs were identified compared with those reported in the published *E. corollae* antenna transcriptome data [6]. Next, we performed phylogenetic analysis using the 40 candidate EcorORs and ORs from *E. balteatus*, *D. melanogaster*, *B. dorsalis* and *C. stygia* (figure 1*a*). The topology of the phylogenetic tree was generally consistent with that obtained in a previous study [6]. The expression levels of *OR* genes in the antenna were higher than those in the proboscis (figure 1*b*). *EcorOrco* had the highest expression level in both tissues. The expression levels of *EcorOR30* in the proboscis were the highest after *EcorOrco*. A volcano plot showed that *EcorOR* genes in the female antenna and proboscis were not significantly differentially expressed between the feeding and starvation groups (figure 2*a*,*b*). By contrast, in males there was significant upregulation of *EcorOR14* and *EcorOR6* gene expression in the feeding group compared with the starvation group, and *EcorOR40* was downregulated in the antennae of males in the feeding group (figure 2*c*). As observed in females, there was no difference in OR gene expression in male proboscises between the feeding and starvation groups (figure 2*d*).

#### 3.2.2. Gustatory receptors

**Figure 3.**
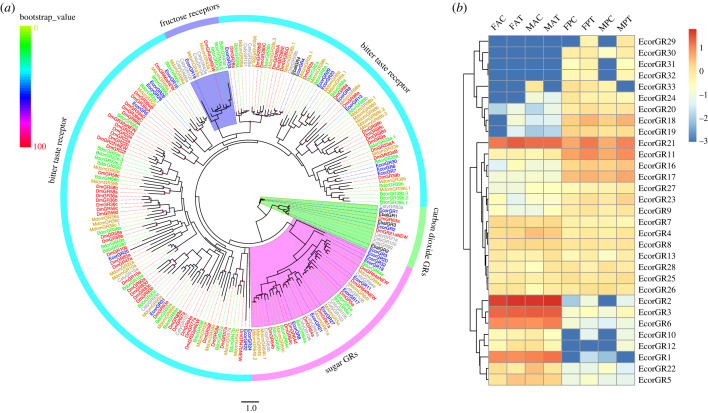
Phylogenetic tree of GRs and heatmap of *GR* expression. (*a*) Phylogenetic tree constructed using GR protein sequences from *E. corollae* (blue), *E. balteatus* (black), *D. melanogaster* (red), *B. dorsalis* (green), *C. stygia* (grey) and *M. domestica* (brown). The phylogenetic tree was rooted using carbon dioxide GR orthologues, and bootstrap values are shown on the left. (*b*) Heatmap of *GR* gene expression levels in the antenna and proboscis. The expression values are represented as mean values, and the data were normalized as follows: log_10_(FPKM + 0.001).

A total of 31 GRs were identified in the antenna and proboscis transcriptomes of *E. corollae*; 17 of these GRs were newly identified and named EcorGR17 to EcorGR33 according to the length of the sequence. Only two newly annotated GRs, EcorGR18 and EcorGR19, had full-length sequences, which contained six to seven transmembrane domains (electronic supplementary material, table S7-1). Phylogenetic analysis revealed that EcorGR8, EcorGR11, EcorGR17, EcorGR18, EcorGR20, EcorGR21, EcorGR27 and EcorGR32 belonged to the sugar receptor subfamily; EcorGR19 clustered with the members of the fructose receptor subfamily [58]. Three EcorGRs, EcorGR1, EcorGR2 and EcorGR3, clustered with the carbon dioxide receptor subfamily. The other GRs were clustered with the bitter taste receptor clade (figure 3*a*).

Of these 31 *GR* genes, four (*EcorGR29*, *EcorGR30*, *EcorGR31* and *EcorGR32*) were only detected in the proboscis, while the others were expressed in both the antennae and proboscis (figure 3*b*). Among them, 11 *GRs* (*EcorGR16*, *EcorGR17*, *EcorGR24*, *EcorGR33*, *EcorGR30*, *EcorGR27*, *EcorGR23*, *EcorGR9*, *EcorGR18*, *EcorGR19* and *EcorGR20*) were expressed at a higher level in the proboscis in both sexes, whereas 8 *GRs* (*EcorGR22*, *EcorGR5*, *EcorGR10*, *EcorGR12*, *EcorGR3*, *EcorGR6*, *EcorGR1* and *EcorGR2*) showed a higher expression level in the antennae compared with the proboscis. Notably, *EcorGR21* was highly expressed in both the antennae and proboscis (figure 3*b*). There was significant upregulation of *EcorGR21* expression in female and male proboscises in the feeding group (figure 2*b*,*d*), but there was no significant difference in expression between the feeding and starvation groups in female and male antennae (figure 2*a*,*c*).

#### 3.2.3. Ionotropic receptors

**Figure 4.**
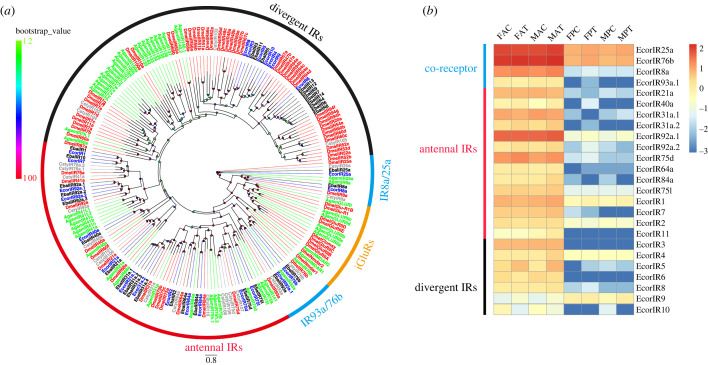
Phylogenetic tree of IRs and heatmap of *IR* expression. (*a*) Phylogenetic tree constructed using IR protein sequences from *E. corollae* (blue), *E. balteatus* (black), *D. melanogaster* (red), *C. stygia* (grey) and *A. gambiae* (green). The IR phylogenetic tree was rooted using IR8a/IR25a orthologues, and bootstrap values are shown on the left. (*b*) Heatmap of *IR* gene expression levels in the antenna and proboscis. The expression values are represented as mean values, and the data were normalized as follows: log_10_(FPKM + 0.001).

A total of 25 IRs were identified in both the antenna and proboscis transcriptomes of *E. corollae*, and four of them (EcorIR8, EcorIR9, EcorIR10 and EcorIR11) were newly identified. The lengths of these IRs ranged from 90 aa to 604 aa, and all were incomplete sequences (electronic supplementary material, table S7-2). A phylogenetic tree was constructed using the IR sequences from different Diptera insects (figure 4*a*). Four EcorIRs (EcorIR8a, EcorIR25a, EcorIR76b and EcorIR93a.1) were clustered into a highly conserved co-receptor subfamily clade. Most of the EcorIRs belonged to a conserved ‘antennal IR’ clade, and seven IRs (EcorIR3, EcorIR4, EcorIR5, EcorIR6, EcorIR8, EcorIR9 and EcorIR10) were clustered into a species-specific ‘divergent IR’ clade (figure 4).

The putative co-receptors *EcorIR76b* and *EcorIR25a* showed the highest expression in both the antenna and proboscis, while the co-receptor *EcorIR8a* was mainly expressed in antennae. Most of the antennal *IRs* were expressed at high levels in antenna, while *EcorIR92a.1*, *EcorIR1* and *EcorIR2* were expressed at relatively high levels in both the antenna and proboscis (figure 4*b*). In male antennae, two *IR* genes, *EcorIR84a* and *EcorIR5*, were significantly upregulated in the feeding group compared with the starvation group (figure 2*c*).

#### 3.2.4. Odorant binding proteins

**Figure 5.**
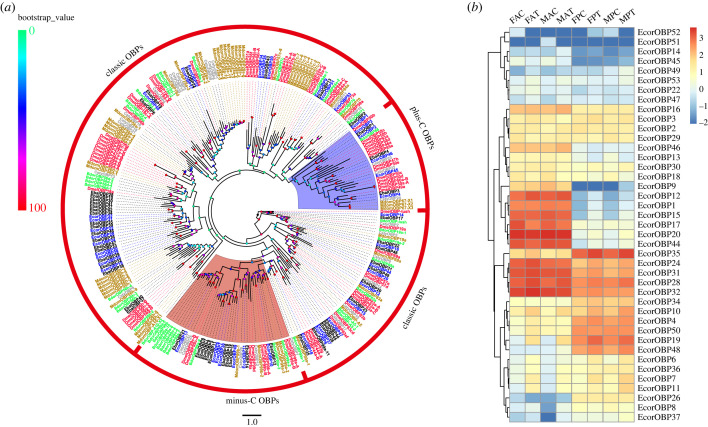
Phylogenetic tree of OBPs and heatmap of *OBP* gene expression. (*a*) Phylogenetic tree of OBPs constructed using proteins from *E. corollae* (blue), *E. balteatus* (black), *D. melanogaster* (red), *B. dorsalis* (green), *C. stygia* (grey) and *M. domestica* (brown). The tree was rooted using Lush-OBP orthologues, and bootstrap values are shown on the left. (*b*) Heatmap of *OBP* gene expression levels in the antenna and proboscis. The expression values are represented as mean values, and the data were normalized as follows: log_10_(FPKM + 0.01).

A total of 41 OBPs were annotated in both the antenna and proboscis transcriptomes of *E. corollae.* Nine *OBPs* were newly identified and were named *EcorOBP45* to *EcorOBP53*. Five of them (*EcorOBP45*, *EcorOBP46*, *EcorOBP48* to *EcorOBP50*) had full-length nucleotide sequences, which encoded proteins ranging from 127 aa to 207 aa and had predicted N-terminal signal peptides (electronic supplementary material, table S7-3). Phylogenetic analysis showed that the EcorOBPs were distributed in different clades. Twenty-nine EcorOBPs were classified into the classical OBP subfamily; these proteins had six conserved cysteines and showed the signature motif C_1_-X_15–39_-C_2_-X_3_-C_3_-X_21–24_-C_4_-X_7–12_-C_5_-X_8_-C_6_ (figure 5*a* and electronic supplementary material, figure S2*a*) [59,60]. Seven EcorOBPs (EcorOBP6, EcorOBP7, EcorOBP10, EcorOBP11, EcorOBP13, EcorOBP36 and EcorOBP49) belonged to the Minus-C OBP subfamily with the signature motif C_1_-X_30_-C_2_-X_38–44_-C_3_-X_18–19_-C_4_ (figure 5*a* and electronic supplementary material, figure S2*b*). The remaining five candidate OBPs (EcorOBP2, EcorOBP3, EcorOBP4, EcorOBP45 and EcorOBP46) had the plus-C gene motif with a predicted signal peptide sequence and a specific C-P-X_9_-C motif at the C terminus (figure 5*a* and electronic supplementary material, figure S2*c*) [61].

*EcorOBPs* exhibited diverse expression patterns in the antennal and proboscis tissues. The *EcorOBP20* gene had the highest expression level in male and female antennae, while *EcorOBP35* was the most highly expressed OBP in the proboscis of both sexes (figure 5*b*). *EcorOBP6*, *EcorOBP10*, *EcorOBP11*, *EcorOBP19* and *EcorOBP50* were upregulated in the antennae of females in the feeding group (figure 2*a*), while *EcorOBP6*, *EcorOBP7*, *EcorOBP19*, *EcorOBP24* and *EcorOBP35* were upregulated in the proboscis of females in the feeding group compared with the starvation group (figure 2*b*). In male antennae, *EcorOBP7*, *EcorOBP10*, *EcorOBP11*, *EcorOBP19* and *EcorOBP36* were upregulated under the feeding treatment compared with the starvation treatment (figure 2*c*), while *EcorOBP6*, *EcorOBP7*, *EcorOBP11* and *EcorOBP48* were upregulated in the male proboscis under feeding treatment (figure 2*d*).

#### 3.2.5. Chemosensory proteins and sensory neuron membrane proteins

**Figure 6.**
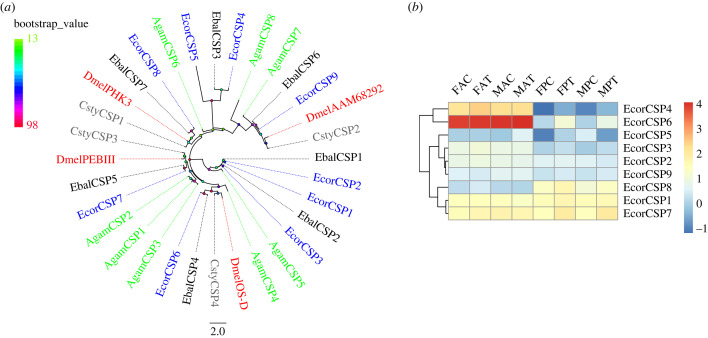
Phylogenetic tree of CSPs and heatmap of *CSP* expression. (*a*) Phylogenetic tree constructed using CSPs from *E. corollae* (blue), *E. balteatus* (black), *D. melanogaster* (red), *C. stygia* (grey) and *A. gambiae* (green). Bootstrap values are shown on the left. (*b*) Heatmap of *CSP* gene expression levels in the antenna and proboscis. The expression values are represented as mean values, and the data were normalized as follows: log_10_(FPKM + 0.01).

A total of nine CSPs were annotated in both the antenna and proboscis transcriptomes of *E. corollae*, and two of them, EcorCSP8 and EcorCSP9, were newly identified. Both of these CSPs had full-length ORFs encoding proteins ranging from 112 aa to 119 aa in length (electronic supplementary material, table S7-4). All CSPs had four conserved cysteine residues (electronic supplementary material, figure S3) [62]. A phylogenetic tree of CSPs was constructed using amino acid sequence from Dipteran species, and a CSP clade specific to the hoverflies *E. corollae* and *E. balteatus* was found (figure 6*a*). *EcorCSP1*, *EcorCSP4*, *EcorCSP6* and *EcorCSP7* in the antenna, and *EcorCSP1*, *EcorCSP6*, *EcorCSP7* and *EcorCSP8* in the proboscis displayed high expression levels in both sexes (figure 6*b*). DEG analysis showed that only *EcorCSP7* in the proboscis was upregulated under feeding treatment compared with starvation treatment, and this upregulation was observed in both males and females (figure 2*d*).

A phylogenetic tree of SNMPs was constructed using the two EcorSNMPs identified from transcriptome analysis and proteins from *A. gambiae*, *E. balteatus* and *D. melanogaste*r (electronic supplementary material, figure S4). In this tree, two EcorSNMPs clustered with single-copy orthologous genes from other Diptera species. Expression analysis showed that *EcorSNMP1* had a higher expression level in the antenna (electronic supplementary material, figure S4). No significantly differentially expressed *EcorSNMPs* were identified (figure 2).

#### 3.2.6. Tissue- and sex-specific expression of differentially expressed genes under different feeding states

**Figure 7.**
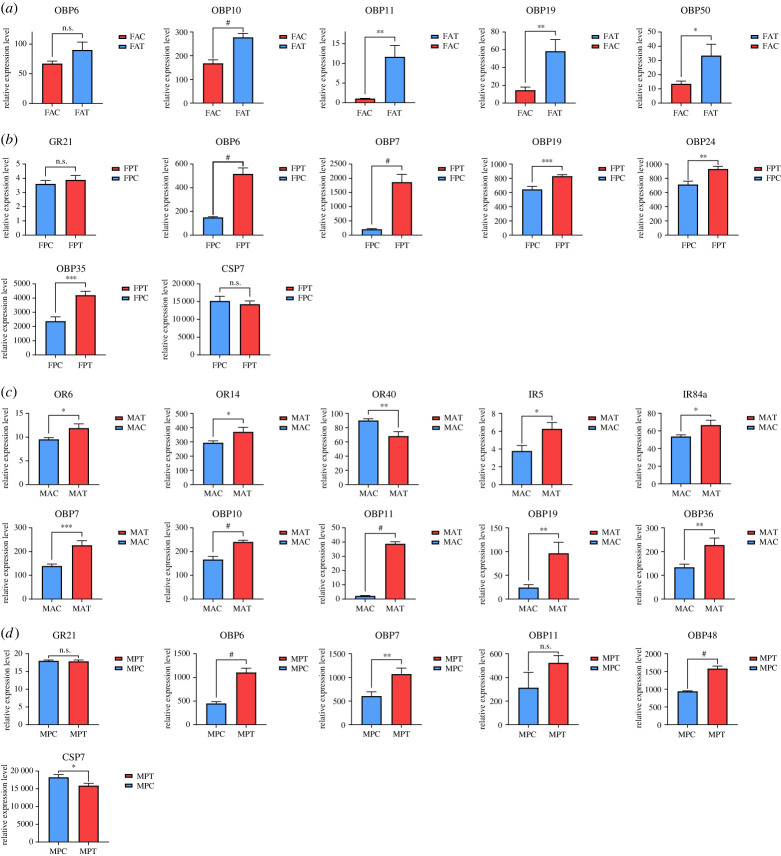
Analysis of differentially expressed chemosensory genes under different feeding states in different tissues of *E. corollae.* (*a–d*) The expression levels of chemosensory-related genes in female antennae (*a*), female proboscises (*b*), male antennae (*c*) and male proboscises (*d*) between the feeding and starvation groups. Data are shown as mean ± s.e.m. (*n* = 3). The asterisks indicate statistically significant differences (independent samples *t*-test, *p < 0.05; **p < 0.01, ***p < 0.001, ^#^*p* < 0.0001), and n.s. indicates no significant difference.

To further verify the differential expression of chemosensory-related genes between the feeding group and starvation group, real-time qPCR was used to determine the expression levels of all 17 chemosensory-related DEGs under different feeding states (figures 2 and 7). The melt curve plots are shown in electronic supplementary material, figure S5, and the standard curves for candidate genes used in this experiment are shown in electronic supplementary material, figure S6. The results showed that most DEGs were upregulated in the feeding group, and the expression of *OBP* genes in the antennae and proboscis was especially impacted by feeding state. For example, *EcorOBP19* in female antennae (figure 7*a*), *EcorOBP6* and *EcorOBP7* in the female proboscis (figure 7*b*), and *EcorOBP11* in male antennae showed much higher expression levels (3-fold higher) in the feeding group compared with the starvation group (*p* < 0.01, figure 7*c*). In addition, some receptor genes, including two *ORs* (*EcorOR6* and *EcorOR14*) and two *IRs* (*EcorIR5* and *EcorIR84a*), in male antennae showed higher expression levels in the feeding group (*p* < 0.05, figure 7*c*). However, the expression levels of *EcorCSP7* in the male proboscis and *EcorOR40* in male antennae were significantly lower in the feeding group (figure 7*c*,*d*). No significant differences in the expression levels of several genes, namely *EcorOBP6* in female antennae, *EcorGR21* in male and female proboscises, *EcorCSP7* in female proboscises, and *EcorOBP11* in male proboscises, were observed between the feeding and starvation groups (*p* > 0.05, figure 7).

## 4. Discussion

In this study, we conducted RNA-seq analysis of the antennae and proboscises of *E. corollae* under different feeding states. We aimed to analyse variations in the expression levels of chemosensory-related genes under different feeding states and investigate whether nutritional state affects olfactory plasticity. By performing de novo transcriptome analysis, we identified 148 candidate chemosensory genes (40 *ORs*, 31 *GRs*, 25 *IRs*, 41 *OBPs*, 9 *CSPs* and 2 *SNMPs*). We found 32 novel chemosensory-related genes not previously reported in the antennal transcriptome of *E. corollae* [6]. The higher number of chemosensory-related genes identified might be attributed to the sequencing of proboscis, as some chemosensory-related genes might have been expressed at low levels or not expressed at all in antennae [6,63,64]. In addition, we identified 16 differentially expressed chemosensory genes through real-time qPCR analysis, confirming the accuracy and reliability of the identified chemosensory-related genes and their expression changes.

Syrphid flies require pollen for ovarian maturation and energy to find mates and lay eggs [29,65–67]. Some chemosensory-related proteins may be involved in sensing the chemical signals from the food and mates. OBPs and CSPs play a crucial role in transporting hydrophobic molecules and facilitating the detection of conspecific partners and food sources [68]. In this study, we identified 41 *EcorOBPs* and 9 *EcorCSPs* from the antennal and proboscis transcriptomes of *E. corollae*. Under different feeding states, the genes encoding these soluble proteins showed significant changes in expression, indicating chemosensory plasticity in syrphid flies. In particular, most of the DEGs were *OBPs*, suggesting their potential roles in post-feeding functions. The expression levels of 10 differentially expressed *EcorOBPs* were upregulated in the feeding treatment group. Similar observations were made in other studies, such as with a study of the predator *Arma chinensis*, which showed that two and nine *AchiOBPs* were upregulated in the female and male antennae, respectively, after feeding [69]. Only *EcorCSP7* was downregulated in the feeding treatment group in our study, consistent with findings in *Glossina morsitans* where the expression levels of *GmorCSP2* increased in females after starvation for 48 h [70,71]. Comparing differential gene expression between males and females in response to feeding, we found that *EcorOBP50* in the antennae and *EcorOBP35* and *EcorOBP24* in the proboscis were specifically upregulated in females after feeding, indicating their potential roles in finding sheltered places or oviposition sites. By contrast, *EcorOBP48* was specifically upregulated and *EcorCSP7* was downregulated in the proboscis after feeding, suggesting that they may be involved in feeding behaviours and gustatory plasticity in males.

In this study, we conducted transcriptome sequencing and analysis of the proboscis in *E. corollae*, an important gustatory organ in adult individuals. Through DEG analysis, we found that the carbon dioxide receptor genes *EcorGR1*, *EcorGR2* and *EcorGR3* exhibited higher expression levels in the antenna compared to the proboscis (figure 3). This suggests that antennae play a crucial role in carbon dioxide detection. We also identified eight *EcorGR* genes as members of the sugar receptor family, including *EcorGR8*, *EcorGR11*, *EcorGR17*, *EcorGR18*, *EcorGR20*, *EcorGR21*, *EcorGR27* and *EcorGR32* (figure 3) [72,73]. These genes may be involved in sugar detection. Notably, EcorGR19 showed homology to the fructose receptor DmelGR43a in *D. melanogaster* [74]. The high expression levels of these sugar receptor genes in the proboscis suggest their crucial roles in taste perception and feeding behaviours.

Several studies have shown that these bitter taste receptors are co-expressed in different combinations and mainly sense compounds such as caffeine, umbelliferone, quinine, denatonium and lobeline, which can be toxic and harmful to insects [58,75–78]. In this study, we also identified candidate bitter taste receptors, including EcorGR5, which is homologous to DmelGR39aA/B in *D. melanogaster*, and EcorGR16, which is homologous to DmelGR66a. Among these candidate bitter taste receptors, we found that *EcorGR29–31* was exclusively detected in proboscis, while *EcorGR5–6* and *EcorGR22* exhibited higher expression level in antennae than in the proboscis. This suggests that these bitter receptors may have a specialized role in sensing bitter compounds in hoverflies. Importantly, we did not observe significant changes in the expression of these *EcorGR* genes between the feeding and starvation treatments, indicating that the nutritional state does not appear to influence the expression levels of *EcorGRs*.

Previous studies have classified the IR family members into two clades [79]. Members of the first clade, known as ‘antennal IRs’, are mainly expressed in the antenna and are involved in the detection of acids, amines and amino acids [48,80–82]. Members of the other clade, referred to as ‘divergent IRs’, are usually expressed in tissues other than the antenna, and recent studies suggest that these receptors may be involved in gustatory perception [83,84]. In this study, we identified seven ‘divergent IRs’. Expression analysis revealed that the ‘antennal IRs’ were highly expressed in the antenna (figure 3*b*), suggesting their roles in the olfactory perception of compounds such as acids or polyamines. The expression levels of the ‘divergent IRs’ *EcorIR4* and *EcorIR9* in the proboscis were the same as those in the antennae, suggesting that they may be involved in both taste and olfaction. Studies in *Drosophila* have demonstrated that DmelIR84a senses food-derived phenylacetic acid and phenylacetaldehyde and is able to regulate male courtship behaviour [85–87]; male *DmelIR84a* mutants showed a reduced electrophysiological response to chemicals and a significant reduction in mating behaviour [87]. In this study, we found that the expression levels of the *EcorIR84a* and *EcorIR5* genes were upregulated in male antennae after feeding. We speculate that the changes in the transcription of *EcorIR84a*, which is related to *DmelIR84a*, may affect male courtship behaviour. Very few functional studies of ‘divergent IRs’ are available, and the potential function of EcorIR5 is still unknown. Hence, further studies are required to investigate its role.

In both females and males, *OR* expression was higher in the antennae than in the proboscis, suggesting that in *E. corollae* ORs are mainly involved in the detecting host volatiles, volatiles from mating partners, volatiles from egg-laying sites and volatiles from food sources. Interestingly, we observed that the expression levels of the individual *EcorOR6* and *EcorOR14* genes in male antennae were upregulated after feeding, in contrast to *ORs* in mosquitoes, which are not typically upregulated after feeding [22]. The discrepancy might be attributed to the distinct habits of mosquitoes and hoverflies. After feeding, hoverflies typically need to search for potential mates and suitable sheltered locations, whereas mosquitoes do not share the same requirements. Based on this observation, we propose that EcorOR6 and EcorOR14 might be involved in recognizing chemical cues related to mates or suitable habitats. Besides, *EcorOR40* expression was downregulated in male antennae in the feeding group relative to the starvation group. The upregulation of some *OR* genes and downregulation of others is similar to the findings of some studies in mosquitoes [22,23], leading us to speculate that this receptor may be involved in the recognition of flower- and nectar-derived chemical cues. Our findings suggest a possible involvement of differentially expressed ORs in the regulation of post-fetching behaviours, such as searching for mates or shelter sites. This hypothesis needs to be verified by further behavioural and receptor function studies.

To summarize, our study successfully identified novel chemosensory genes from the antennal and proboscis transcriptomes of *E. corollae*. Furthermore, we examined the expression profile changes of chemosensory-related genes across different tissues and feeding conditions. Particularly, we observed that the expression levels of certain *EcorOBP*, *EcorCSP*, *EcorOR* and *EcorIR* genes were influenced by the feeding status. These findings provide a valuable molecular foundation for comprehending the olfactory mechanisms involved in feeding and post-feeding physiological activities of *E. corollae*.

## Data Availability

The raw data for the study can be accessed at BioProject PRJNA791698 (https://www.ncbi.nlm.nih.gov/bioproject/PRJNA791698). Supplementary material is available online [88].
